# Differences in GlycA and lipoprotein particle parameters may help distinguish acute kawasaki disease from other febrile illnesses in children

**DOI:** 10.1186/s12887-016-0688-5

**Published:** 2016-09-05

**Authors:** Margery A. Connelly, Chisato Shimizu, Deborah A. Winegar, Irina Shalaurova, Ray Pourfarzib, James D. Otvos, John T. Kanegaye, Adriana H. Tremoulet, Jane C. Burns

**Affiliations:** 1 LipoScience, Laboratory Corporation of America® Holdings, 2500 Sumner Blvd, Raleigh, NC 27616 USA; 2 Department of Pediatrics, University of California, San Diego, CA USA; 3 Rady Children’s Hospital, San Diego, CA USA

**Keywords:** Kawasaki disease, Biomarkers, GlycA, Lipoprotein particle number

## Abstract

**Background:**

Glycosylation patterns of serum proteins, such as α1-acid glycoprotein, are modified during an acute phase reaction. The response of acute Kawasaki disease (KD) patients to IVIG treatment has been linked to sialic acid levels on native IgG, suggesting that protein glycosylation patterns vary during the immune response in acute KD. Additionally, the distribution and function of lipoprotein particles are altered during inflammation. Therefore, the aim of this study was to explore the potential for GlycA, a marker of protein glycosylation, and the lipoprotein particle profile to distinguish pediatric patients with acute KD from those with other febrile illnesses.

**Methods:**

Nuclear magnetic resonance was used to quantify GlycA and lipoprotein particle classes and subclasses in pediatric subjects with acute KD (*n* = 75), post-treatment subacute (*n* = 36) and convalescent (*n* = 63) KD, as well as febrile controls (*n* = 48), and age-similar healthy controls (*n* = 48).

**Results:**

GlycA was elevated in acute KD subjects compared to febrile controls with bacterial or viral infections, IVIG-treated subacute and convalescent KD subjects, and healthy children (*P* <0.0001). Acute KD subjects had increased total and small low density lipoprotein particle numbers (LDL-P) (*P* <0.0001) and decreased total high density lipoprotein particle number (HDL-P) (*P* <0.0001) compared to febrile controls. Consequently, the ratio of LDL-P to HDL-P was higher in acute KD subjects than all groups tested (*P* <0.0001). While GlycA, CRP, erythrocyte sedimentation rate, LDL-P and LDL-P/HDL-P ratio were able to distinguish patients with KD from those with other febrile illnesses (AUC = 0.789–0.884), the combinations of GlycA and LDL-P (AUC = 0.909) or GlycA and the LDL-P/HDL-P ratio (AUC = 0.910) were best at discerning KD in patients 6–10 days after illness onset.

**Conclusions:**

High levels of GlycA confirm enhanced protein glycosylation as part of the acute phase response in KD patients. When combined with common laboratory tests and clinical characteristics, GlycA and NMR-measured lipoprotein particle parameters may be useful for distinguishing acute KD from bacterial or viral illnesses in pediatric patients.

**Electronic supplementary material:**

The online version of this article (doi:10.1186/s12887-016-0688-5) contains supplementary material, which is available to authorized users.

## Background

Kawasaki disease (KD) is a self-limited vasculitis that typically presents in young children as an acute illness with fever and mucocutaneous changes [1, 2]. If KD is incorrectly diagnosed or left untreated, coronary artery aneurysms (CAA) may develop, predisposing KD patients to long-term cardiovascular complications including myocardial ischemia and infarction [3, 4]. Therefore, early diagnosis and treatment in the acute phase of the disease with intravenous immunoglobulin (IVIG) are important to reduce the risk of CAA [5, 6]. Diagnosis of KD, however, is challenging because the clinical signs are shared by other childhood acute rash/fever illnesses and there are no laboratory tests with sufficient specificity or sensitivity for detection of KD [7]. Therefore, additional biomarkers are needed to aid in diagnosis.

GlycA is a novel, nuclear magnetic resonance (NMR)-measured marker of inflammation whose signal arises from the carbohydrate side-chains of circulating proteins [8]. Hence, GlycA is a composite biomarker that integrates both the protein levels and glycosylation states of the most abundant acute phase proteins in serum. GlycA predicted adverse cardiovascular events in subjects in the Women’s Health, JUPITER, PREVEND, CATHGEN and Intermountain Heart Collaborative studies, independent of traditional risk factors [9–13]. GlycA also predicted incident diabetes in a population of apparently healthy US women as well as in a large population of men and women in the Netherlands [14, 15]. Elevated GlycA was shown to be associated with disease activity and coronary atherosclerosis in rheumatoid arthritis patients [16]. Little is known, however, about the levels of this novel biomarker in acute pediatric illnesses such as KD. Glycosylation patterns of serum and cell surface proteins are critical determinants of the immune response [17, 18], and response of acute KD patients to IVIG treatment has been linked to sialic acid levels on native IgG [19]. We, therefore, hypothesized that GlycA levels are elevated in pediatric subjects with acute KD and serve as an indicator of acute inflammation.

Recently we reported that pediatric and adult KD subjects evaluated during the late convalescent phase of their illness had a similar lipoprotein profile compared to age-matched, healthy controls [20]. However, there are specific changes in the distribution of lipoprotein particle classes and subclasses in patients with active chronic inflammatory disease [21, 22]. Therefore we posited that the severe systemic inflammation that occurs in acute KD modifies the lipoprotein particle distribution and that these alterations may serve as additional markers of KD.

The aim of this study was to determine the ability of GlycA concentrations and NMR lipoprotein particle measures to distinguish pediatric patients with acute KD from those with other febrile illnesses.

## Methods

### Study population

Subjects included 75 children diagnosed with KD according to American Heart Association (AHA) criteria and treated at Rady Children’s Hospital in San Diego, CA between November 2005 and June 2011. Specimens were obtained during 4 stages of KD: 1) pre-IVIG treatment acute (2–10 days after start of illness; illness day 1 defined as first day of fever), and post-IVIG treatment 2) subacute (13–24 days after start of illness), 3) early convalescent (25–75 days after start of illness) and 4) late convalescent (9–49 months after start of illness). The post-treatment subacute, early and late convalescent KD samples were obtained from subjects enrolled during the acute phase of illness. However, not all subjects provided samples during each of the subsequent stages.

Coronary artery status for acute KD subjects was classified as dilated if the internal diameter normalized for body surface area (Z score) was equal to or greater than 2.5 standard deviation units from the mean for the left anterior descending or right coronary arteries assessed by echocardiography during the first 6 weeks after disease onset. Aneurysms were defined as segments ≥1.5 times the diameter of the adjacent segments. Resistance to IVIG treatment was defined as fever at ≥36 h after the end of the IVIG infusion [7].

Forty-eight age-similar, healthy children undergoing minor orthopedic surgical procedures and 48 febrile children with acute illnesses of viral (adenoviral infection, influenza A or B, and viral syndrome) or bacterial (scarlet fever, cutaneous abscess) origin were also included in the study as healthy or febrile controls (Table 1). Written informed consent and assent when appropriate were obtained from the parents and subjects, respectively. The protocol was approved by the Institutional Review Board at the University of California San Diego.

**Table 1:** Clinical characteristics of pediatric untreated acute and IVIG-treated subacute and convalescent KD subjects and healthy and febrile controls

Characteristic	Acute KD 2–10 days (*n* = 75)	Subacute KD 13–24 days (*n* = 36)	Early Convalescent KD 25–75 days (*n* = 43)	Late Convalescent KD 9–49 months (*n* = 20)	Acute Febrile Controls Bacterial origin (*n* = 12)	Acute Febrile Controls Viral origin (*n* = 36)	Healthy Controls (*n* = 48)
Male, *n* (%)	39 (52)	20 (56)	26 (60)	11 (55)	10 (83)	21 (58)	–
Age, years	3.6 (1.8–5.0)	2.9 (1.7–4.6)	3.1 (1.7–4.5)	8.0 (6.7–9.2)	6.2 (4.7–8.2)	2.8 (1.6–5.3)	4.7 (3.0–6.6)
Illness day/month	6.0 d (4.5–7.0)	19 d (16–21)	48 d (34–55)	16 m (14–29)	3.5 d (3.0–4.3)	6.0 d (4.0–6.3)	–
Median lab values							
WBC, ×10^3^/μL	13.7 (10.6–17.3)	7.3 (6.8–9.0)	7.4 (6.0–8.5)	–	12.4 (10.1–14.5)#	8.7 (6.3–11.5)#	–
PMN, %	56 (45–66)	37 (26–50)	37 (27–42)	–	62 (46–70)	40 (23–54)	–
ANC, cells/μL	9646 (6693–12,131)	3025 (1953–3950)	2516 (1823–3525)	–	7014 (5565–9444)	3408 (2071–6764)	–
ESR, mm/h	61 (47–75)	37 (31–57)	17 (9–31)	–	31 (22–40)#	30 (17–39)#	–
CRP, mg/dL	7.4 (4.7–16.7)	0.3 (0.3–0.8)	0.3 (0.3–0.3)	–	2.2 (1.0–8.6)#	4.1 (2.2–5.0)#	–
GlycA, μmol/L	808 (693–919)	440 (382–512)	309 (272–377)	341 (319–395)	595 (571–745)	611 (551–702)	319 (290–354)

Median (IQR = Interquartile Range; 25th-75th percentile); *KD* Kawasaki Disease, *d* days, *m* months, *WBC* white blood cell count, *PMN* polymorphonuclear cells, *ANC* absolute neutrophil count, *ESR* erythrocyte sedimentation rate, *CRP* C-reactive protein; GlycA, NMR-measured marker of systemic inflammation. #Lab data available for WBC 46 subjects, ESR 42 subjects, CRP 31 subjects

Acute KD samples were taken pre-IVIG treatment. All other KD samples were taken post-IVIG treatment

### Inflammatory markers

White blood cell count (WBC), absolute neutrophil count (ANC), percent polymorphonuclear cells (PMN), high sensitivity C-reactive protein (CRP), and erythrocyte sedimentation rate (ESR) were measured as part of clinical care.

### NMR analysis

NMR spectra were acquired from EDTA plasma samples as previously described for the *NMR LipoProfile®* (lipoprotein particle) test at LipoScience (now Labcorp, Raleigh, NC) [23]. The NMR Profiler platform is comprised of a 9.4T (400 MHz ^1^H frequency) spectrometer (Bruker Biospin) with an integrated fluidics sample delivery system. The GlycA signal was quantified as previously described [8]. Lipoprotein particle profiles, triglycerides (TG) and high density lipoprotein cholesterol (HDL-C) were quantified using the LipoProfile-3 algorithm. Briefly, lipoprotein particle classes [very low density lipoprotein (VLDL), low density lipoprotein (LDL) and high density (HDL)] and subclasses [small, medium and large] were quantified using the amplitudes of their spectroscopically distinct lipid methyl group NMR signals [23]. Quantification of TG and HDL-C was accomplished by converting NMR particle numbers to lipid mass concentration units, assuming that the lipoprotein particles have normal lipid content. Previously published data showed that NMR-derived TG and HDL-C values correlate well with chemically measured values [23]. Samples were collected, stored at −80 °C and run in batch in 2014. Stability of GlycA and the lipoprotein analytes in frozen samples are stable for at least 6.5 years when stored at −80 °C [8, 23].

### Statistical analysis

Patient characteristics were summarized by group. Medians and interquartile ranges (IQRs) were reported for continuous variables, and frequency counts and percentages were reported for categorical variables. One-way ANOVA and Dunnet’s multiple comparison were used to compare the differences between groups, and two-tailed *P* values <0.05 were considered statistically significant. Spearman rank correlation coefficients (r) were calculated to evaluate interrelations between the inflammatory markers and lipoprotein parameters, and Mann–Whitney tests were used to gauge the significance of the association. Statistical analyses were performed using GraphPad Prism version 6. To assess the performance of these biomarkers as potential diagnostic tools, receiver operating characteristic (ROC) areas under the curve (AUC), likelihood ratios, positive predictive values, negative predictive values and Wald (*χ*^2^) or z-statistics were calculated using Excel Analyse-it® v3.9.01.

## Results

**Fig. 1:**
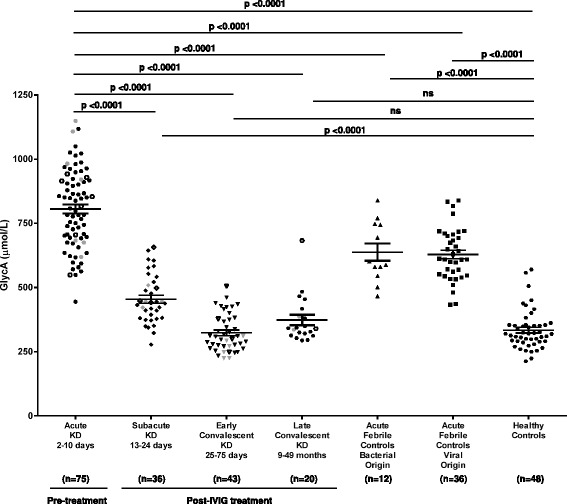
Plasma concentrations of GlycA (μmol/L) among patients with: acute, subacute, early and late convalescent KD patients, febrile controls of bacterial and viral origin, and healthy controls. KD subjects are identified by coronary artery status: normal acute echocardiogram (*solid black symbol*); CAA (*open symbol*); or dilated coronary artery (*solid gray symbol*). *Horizontal bars* represent median and interquartile range

Clinical characteristics of treatment naïve pediatric acute KD subjects (*n* = 75) as well as IVIG-treated subjects with subacute (13–24 days; *n* = 36), early convalescent (25–75 days; *n* = 43) and late convalescent KD (9–49 months; *n* = 20), are described in Table 1 along with age-similar healthy pediatric controls (*n* = 48) and pediatric subjects with various acute febrile illnesses of bacterial (*n* = 12) and viral (*n* = 36) origin. Although GlycA levels were similar between healthy controls and convalescent KD patients, levels were higher in children with acute febrile illness and subacute KD than in healthy controls (*P* <0.0001), or convalescent KD subjects (*P* <0.0001) (Table 1, Fig. 1) and further elevated in acute KD patients (*P* <0.0001). Approximately one third of the subjects with acute KD had higher GlycA concentrations than any of the febrile subjects with acute illness (Fig. 1). GlycA concentrations were higher in subjects with acute KD than in those with influenza A or B, scarlet fever or viral syndrome (*P* <0.0001, 0.01 and 0.0001, respectively) but not in subjects with abscesses or adenoviral infections (Additional file 1: Figure S1). GlycA levels did not differ between acute KD patients who were resistant and those who responded to IVIG treatment (Additional file 2: Figure S2) or between acute KD subjects with CAA or dilated coronary arteries and those with normal echocardiograms (Fig. 1 and Additional file 2: Figure S2).

GlycA was strongly correlated with inflammatory markers such as WBC (*r* = 0.43), ANC (*r* = 0.42) and ESR (*r* = 0.42) (all *P* <0.001) and weakly correlated with CRP (*r* = 0.26, *P* <0.05) in acute KD patients (Additional file 3: Table S1). In addition, GlycA correlated strongly with illness day (*r* = 0.48, *P* <0.0001), while CRP was inversely correlated with this measure (*r* = −0.35, *P* <0.01) (Additional file 3: Table S1).

**Fig. 2:**
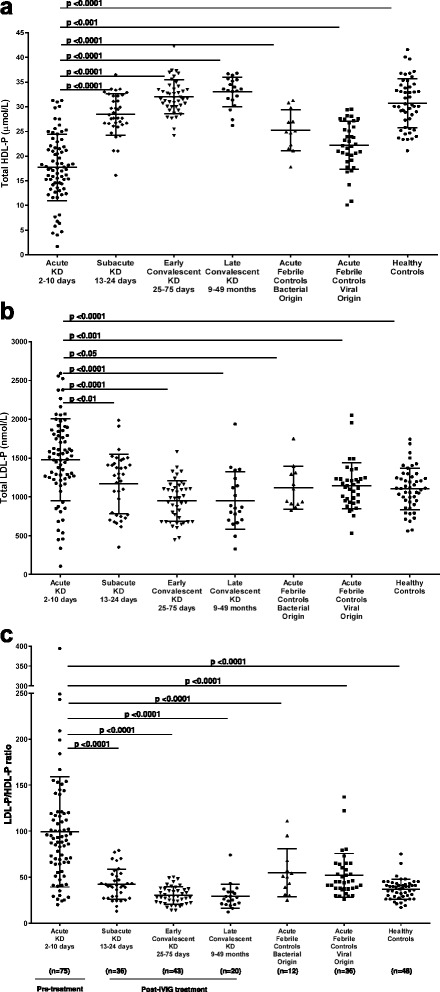
**a) ** Total HDL-P, **b**) total LDL-P and **c**) the ratio of total LDL-P to total HDL-P among acute, subacute, early and late convalescent KD patients, febrile controls of bacterial and viral origin, and healthy controls. *Horizontal bars* represent median and interquartile range

Analysis of NMR-measured lipoprotein and lipid parameters revealed that TG and TG-rich lipoprotein particles (VLDL measures) did not differ between healthy controls, pediatric KD patients and children with acute febrile illness (Table 2). There were, however, notable differences in HDL-P, LDL-P (Fig. 2a and b) and their subclasses among the various groups. Acute KD subjects and febrile controls had decreased HDL-C and total HDL-P (*P* <0.0001). Acute KD subjects and children with viral illnesses had decreased large HDL-P (*P* <0.0001), while acute KD subjects and children with bacterial illnesses had reduced small HDL-P (*P* <0.0001) compared to healthy controls and convalescent KD subjects (Table 2). Subjects with acute KD had higher total and small LDL-P (*P* <0.0001) than subjects in other groups. In addition, they had lower large LDL-P, leading to a reduction in weighted-average LDL size, compared to all groups except those with acute febrile illness of viral origin (data not shown). Subjects with subacute KD had lipoprotein profiles between those of acute and convalescent illness, with HDL and LDL parameters nearing those of healthy controls (Table 2). Subjects with subacute, early and late convalescent KD had LDL-P/HDL-P ratios that mirrored those of normal healthy controls (Table 2). The LDL-P/HDL-P ratio was higher in acute KD subjects than in the other six groups (*P* <0.0001), while no significant differences were observed among healthy controls, subacute and convalescent KD subjects, and febrile controls (Fig. 2c).

**Table 2:** Lipid profiles for pediatric untreated acute and IVIG-treated subacute and convalescent KD subjects and healthy and febrile pediatric controls

Characteristic	Acute KD 2–10 days (*n* = 75)	Subacute KD 13–24 days (*n* = 36)	Early Convalescent KD 25–75 days (*n* = 43)	Late Convalescent KD 9–49 months (*n* = 20)	Acute Febrile Controls Bacterial origin (*n* = 12)	Acute Febrile Controls Viral origin (*n* = 36)	Healthy Controls (*n* = 48)
HDL-C, mg/dL	26 (19–33)	44 (39–47)	53 (48–58)	56 (52–65)	37 (32–41)	34 (28–39)	49 (42–54)
TG, mg/dL	78 (66–97)	104 (83–142)	79 (67–105)	68 (65–99)	86 (55–120)	63 (56–75)	93 (73–115)
LDL particles, nmol/L							
Total LDL-P	1513 (1228–1826)	1259 (766–1416)	984 (680–1115)	861 (705–1167)	1186 (934–1273)	1052 (916–1231)	1110 (948–1248)
IDL-P	110 (65–159)	128 (56–176)	100 (44–143)	88 (63–118)	88 (65–117)	78 (55–107)	106 (71–145)
Large LDL-P	116 (29–244)	472 (261–629)	415 (281–497)	339 (290–527)	299 (216–340)	99 (47–215)	558 (426–718)
Small LDL-P	1230 (880–1574)	483 (392–677)	435 (331–532)	456 (291–536)	754 (549–973)	809 (688–996)	394 (190–531)
HDL particles, μmol/L							
Total HDL-P	18 (14–22)	28 (27–32)	32 (30–34)	33 (32–35)	23 (22–24)	23 (20–27)	30 (27–34)
Large HDL-P	2.1 (1.4–3.1)	4.4 (3.3–5.4)	5.9 (4.6–7.6)	6.2 (4.8–8.5)	4.0 (2.8–6.2)	2.2 (1.3–3.2)	4.7 (3.8–5.8)
Medium HDL-P	8.6 (4.7–12.8)	11.4 (9.8–13.9)	11.7 (10.6–14.5)	12.4 (9.6–14.2)	12.9 (9.0–15.9)	10.2 (7.9–12.9)	9.1 (5.8–12.7)
Small HDL-P	6.8 (3.9–9.2)	11.8 (9.5–13.5)	13.6 (12.0–15.0)	13.9 (11.5–15.8)	4.5 (2.6–7.4)	10.5 (7.1–13.1)	16.2 (14.4–18.0)
VLDL particles, nmol/L							
Total VLDL-P	39 (23–62)	56 (30–82)	36 (24–63)	31 (23–56)	57 (21–75)	27 (19–35)	49 (31–69)
Large VLDL-P	0.9 (0.6–1.7)	1.9 (1.2–5.0)	1.2 (0.8–2.1)	1.1 (0.6–1.4)	0.9 (0.4–1.5)	1.3 (0.9–1.5)	1.4 (0.9–2.8)
Medium VLDL-P	4.5 (1.8–10.4)	15.3 (7.3–28.7)	8.8 (3.2–16.5)	5.2 (0.6–1.4)	3.7 (2.5–18.2)	4.1 (2.0–8.1)	9.1 (4.6–19.4)
Small VLDL-P	31 (18–52)	29 (15–45)	31 (18–42)	26 (17–41)	46 (17–57)	19 (12–29)	35 (21–48)
LDL-P/HDL-P	92 (65–119)	41 (28–51)	29 (22–37)	27 (21–34)	50 (37–59)	45 (37–61)	38 (29–42)

Median (IQR = Interquartile Range; 25th-75th percentile). *LDL-C* low density lipoprotein cholesterol, *HDL-C* high density lipoprotein cholesterol, *TG* triglycerides, *LDL-P* low density lipoprotein particle number, *IDL-P* intermediate density lipoprotein particle number, *HDL-P* high density lipoprotein particle number, *VLDL-P* very low density particle number. Acute KD samples were taken pre-IVIG treatment. All other KD samples were taken post-IVIG treatment

**Fig. 3:**
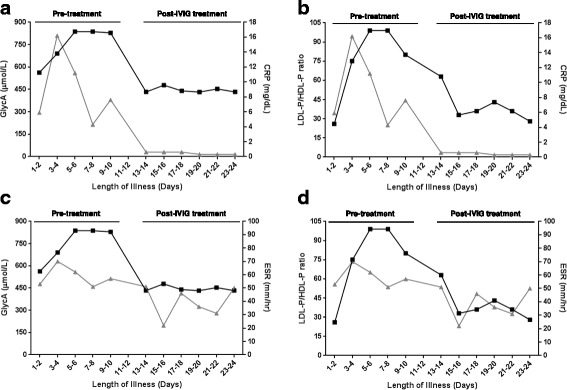
Time course of biomarkers (median) in KD subjects from acute phase to early convalescence: **a**) GlycA (left y-axis; *black line* and *symbols*) and CRP (right y-axis; *dark gray line* and *symbols*), **b**) LDL-P/HDL-P ratio (left y-axis; *black line* and *symbols*) and CRP (right y-axis; *dark gray line* and *symbols*), **c**), GlycA (left y-axis; *black line* and *symbols*) and ESR (right y-axis; *dark gray line* and *symbols*), **d**) LDL-P/HDL-P ratio (left y-axis; *black line* and *symbols*) and ESR (right y-axis; *dark gray line* and *symbols*) in KD patients plotted as a function of illness day (Illness day 1 = first day of fever). Illness day 1 = first day of fever. Pre-IVIG treatment = days 2–10 and post-IVIG treatment = days 13–24

To further explore the relationships between GlycA, CRP, ESR and the ratio of LDL-P/HDL-P with time, we plotted each biomarker as a function of illness day (Fig. 3). CRP peaked on day 3 and trended downward to approximately 50 % of peak levels by days 5–6, whereas GlycA continued to rise until days 5–6 and remained high through days 9–10 (Fig. 3a). Similar to GlycA, the LDL-P/HDL-P ratio continued to rise until days 7–8 and remained high until after IVIG-treatment (Days 13–15, Fig. 3b). ESR rose early and remained high, but exhibited a high degree of variability (Fig. 3c and d). Notably, GlycA and the LDL-P/HDL-P ratio appeared to distinguish themselves from the other biomarkers between days 6 and 10.

**Fig. 4:**
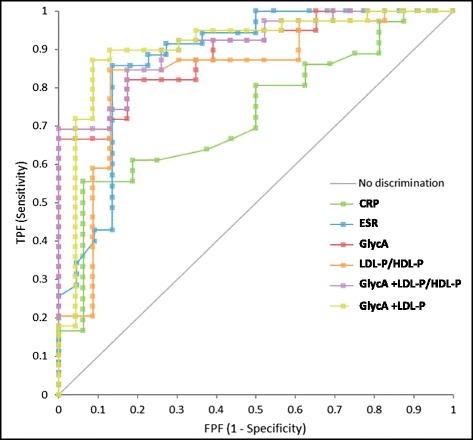
ROC curves for the discrimination of KD from febrile illnesses during illness days 6–10 by CRP, ESR, GlycA, LDL-P/HDL-P, GlycA and LDL-P/HDL-P and GlycA + LDL-P

A ROC analysis was performed to evaluate the relative strengths of the NMR-measured biomarkers, CRP and ESR, at differentiating acute KD from combined febrile illness (bacterial and viral origin). The highest ROC AUCs (95 % confidence intervals) were 0.789 (0.707–0.872), 0.820 (0.721–0.918), 0.823 (0.752–0.894) and 0.884 (0.814–0.954) for the LDL-P/HDL-P ratio, CRP, GlycA and ESR, respectively, for illness days 2–10. A ROC analysis restricted to illness days 6–10 revealed AUC values of 0.691 (0.556–0.826), 0.730 (0.587–0.873), 0.842 (0.732–0.952), O.864 (0.765–0.963), 0.879 (0.778–0.979) and 0.891 (0.814–0.967), for total HDL-P, CRP, the LDL-P/HDL-P ratio, total LDL-P, ESR and GlycA, respectively (Additional file 4: Table S2). GlycA added to total LDL-P or the LDL-P/HDL-P ratio, resulted in AUCs (95 % confidence interval) of 0.909 (0.823–0.994) and 0.910 (0.838–0.982), respectively (Additional file 4: Table S2). The Wald test or z-statistics were 3.15, 6.09, 7.22, 7.37, 9.40, 9.99 and 11.65, and the positive likelihood ratios were 8.89, 6.49, 14.15, 6.29, 16.51, 15.33 and 15.92 for CRP, the LDL-P/HDL-P ratio, total LDL-P, ESR, GlycA plus total LDL-P, GlycA and GlycA plus LDL-P/HDL-P, respectively (Additional file 4: Table S2). A comparison of the ROC curves for all 6 biomarkers can be found in Fig. 4.

## Discussion

In this study, we identified changes in NMR-measured lipoprotein particle parameters and GlycA that may be useful in diagnosing acute KD or in monitoring resolution of inflammation after treatment. GlycA levels were higher in children with acute KD than in those with other febrile illnesses. GlycA levels in acute KD subjects were also higher than levels observed in subjects with chronic inflammatory-mediated illnesses (e.g. cardiovascular disease, rheumatoid arthritis and systemic lupus erythematosus) [9–16, 24]. In addition, lipoprotein particle class and subclass numbers differed between patients with acute KD and those with acute febrile illnesses. Several HDL-P measures were lower in acute KD patients than in healthy, convalescent KD and febrile controls. Total LDL-P and the ratio of LDL-P to HDL-P were higher in acute KD patients than in the other control groups. These changes are consistent with previous observations that acute phase proteins (e.g. α1-acid glycoprotein, serum amyloid A, ceruloplasmin) and lipases (e.g. endothelial lipase, secreted phospholipase A_2_-IIa and -V), which are upregulated during inflammation, modify the size distribution and function of lipoprotein particles [25–29].

During acute inflammation, serum levels of CRP and serum amyloid A (SAA) rise and fall within the first few days [30]. In contrast, serum levels of the proteins that give rise to the GlycA NMR signal (e.g. α1-acid glycoprotein, haptoglobin, α1-antitrypsin, α1-antichymotrypsin) rise more slowly, reaching peak concentrations several days after onset of an acute inflammatory reaction, and fall slowly over weeks [30]. Our data support these observations, as CRP levels rose early and fell by day 6 while GlycA levels rose later and remained high through day 10. The finding that GlycA exhibited a strong correlation with illness day, while CRP showed an inverse correlation confirms the results of the kinetic data. Therefore, GlycA and CRP levels may add independent value when evaluating patients with inflammatory illnesses such as KD; especially 6–10 days after illness onset when levels of these two biomarkers varied in relation to one another.

The ROC AUCs of 0.910 and 0.909 for GlycA combined with either the LDL-P/HDL-P ratio or LDL-P, respectively, suggest that these biomarker combinations are better at distinguishing KD from other febrile illnesses than CRP, ESR or any of the 3 biomarkers alone. The results of the Wald and Likelihood Ratio tests, alternative measures of the performance of a diagnostic test, suggest that the combinations of GlycA with LDL-P/HDL-P or LDL-P will correctly distinguish patients with KD from those with febrile illnesses more often than CRP or ESR alone. Thus, NMR-measured biomarkers may be useful in determining antecedent inflammation in patients presenting later (6–10 days) in the course of acute KD. These biomarkers may also be useful in patients whose fever and other mucocutaneous signs have resolved but for whom subsequent periungual peeling prompts evaluation for missed KD. Unfortunately we were unable to test this hypothesis with our data, given that all subjects with acute KD received IVIG-treatment by 10 days after fever onset. Therefore, future studies with samples taken from patients untreated beyond 10 days of fever onset will more accurately determine the kinetics of GlycA and LDL-P/HDL-P ratio as markers of inflammation and will address the question of whether or not these biomarkers will be useful for missed KD.

When used along with the typical clinical characteristics observed in patients with pediatric febrile illnesses and standard diagnostic tools, the combination of GlycA with either LDL-P or the LDL-P/HDL-P ratio may help identify children with KD. GlycA has the advantage of being a more stable biomarker, in terms of within-subject biological variation, than CRP [8]. To illustrate this point we determined the homeostatic set points, the true values of analytes in biological homeostasis, for CRP and GlycA in a cohort of normal, healthy, adult volunteers (*n* = 23) collected for a previously published biological variability study [8]. We calculated that 33 concurrent measurements of CRP would be required to estimate its true value for a single patient, compared to only one for GlycA [8, 31, 32]. In addition, while the ESR is affected by IVIG treatment, GlycA is unaffected by the presence of IgG [8] or IVIG [unpublished results]. Therefore, the combinations of GlycA with either LDL-P/HDL-P or LDL-P are may provide an additional tool for pediatric patient care.

We recognize both strengths and weaknesses in our study. This is the first study to assess glycosylation levels of plasma proteins in a well-characterized cohort of KD and febrile controls who share some clinical features with acute KD patients. However, sample size was limited and would need to be increased in independent cohorts of pediatric subjects in order to test the predictive value of these biomarkers in KD diagnosis. In addition, the kinetic analysis suffered by the limitation that the samples were not taken from the same subjects at each time point. Finally, the study would have benefited by the inclusion of additional samples from patients with scarlet fever, adenoviral infection and juvenile idiopathic arthritis as these illnesses are most likely to cause clinical confusion with KD. Therefore future studies will include samples from these illnesses as well as serious illnesses such as bacterial pneumonia, pyelonephritis and sepsis.

## Conclusions

High levels of GlycA confirm enhanced protein glycosylation as part of the acute phase response in KD patients. When combined with common laboratory tests and clinical characteristics, GlycA and NMR-measured lipoprotein particle parameters may be useful for distinguishing acute KD from bacterial or viral illnesses in pediatric patients.

## Additional files

Additional file 1: Figure S1. Plasma concentrations of GlycA (μmol/L) in subjects with: acute KD, abscess, adenoviral infection, influenza A or B, scarlet fever or viral syndrome. ns = not statistically significant. Horizontal bars represent median and interquartile range. (PPTX 70 kb) Additional file 2: Figure S2. Plasma concentrations of GlycA (μmol/L) in acute KD patients who responded to intravenous immunoglobulin (IVIG) treatment, those who were IVIG-resistant, and healthy controls. Noted are acute KD subjects with a normal echocardiogram (solid black symbol) as well as those who developed a CAA (open symbol) or a dilated coronary artery (solid gray symbol). ns = not statistically significant. Horizontal bars represent median and interquartile range. (PPTX 58 kb) Additional file 3: Table S1. Correlations among inflammatory markers in acute KD subjects. (DOCX 15 kb) Additional file 4: Table S2. SPerformance characteristics for GlycA, lipoprotein parameters, CRP and ESR restricted to illness days 6-10. (DOCX 23 kb)

## Abbreviations

ANC: Absolute neutrophil count
AUC: Area under the curve
CAA: Coronary artery aneurysm
CATHGEN: CATHeterization GENetics registry
CRP: C-reactive protein
ESR: Erythrocyte sedimentation rate
HDL-C: High density lipoprotein cholesterol
HDL-P: High density lipoprotein particle number
IQR: Interquartile range
IVIG: Intravenous immunoglobulin
JUPITER: Justification for the use of statins in primary prevention: an intervention trial
KD: Kawasaki disease
LDL-P: Low density lipoprotein particle number
NMR: Nuclear magnetic resonance
PMN: Polymorphonuclear cells
PREVEND: Prevention of renal and vascular end-stage disease study
ROC: Receiver operating characteristic
TG: Triglycerides
VLDL: Very low density lipoprotein
WBC: White blood cell count
WHS: Womens Health Study

## References

[1] Kawasaki T, Kosaki F, Okawa S, Shigematsu I, Yanagawa H (1974). A new infantile acute febrile mucocutaneous lymph node syndrome (MLNS) prevailing in Japan. Pediatrics.

[2] Burns JC, Glode MP (2004). Kawasaki syndrome. Lancet.

[3] Suzuki A, Kamiya T, Kuwahara N, Ono Y, Kohata T, Takahashi O (1986). Coronary arterial lesions of Kawasaki disease: cardiac catheterization findings of 1100 cases. Pediatr Cardiol.

[4] Gordon JB, Kahn AM, Burns JC (2009). When children with Kawasaki disease grow up: myocardial and vascular complications in adulthood. JACC.

[5] Ogata S, Tremoulet AH, Sato Y, Ueda K, Shimizu C, Sun X (2013). Coronary artery outcomes among children with Kawasaki disease in the United States and Japan. Int J Cardiol.

[6] Newburger JW, Takahashi M, Beiser AS, Burns JC, Bastian J, Chung KJ (1991). A single intravenous infusion of gamma globulin as compared with four infusions in the treatment of acute Kawasaki syndrome. NEJM.

[7] Newburger JW, Takahashi M, Gerber MA, Gewitz MH, Tani LY, Burns JC (2004). Diagnosis, treatment, and long-term management of Kawasaki disease: a statement for health professionals from the Committee on Rheumatic Fever, Endocarditis and Kawasaki Disease, Council on Cardiovascular Disease in the Young, American Heart Association. Circ.

[8] Otvos JD, Shalaurova I, Wolak-Dinsmore J, Connelly MA, Mackey RH, Stein JH (2015). GlycA: a composite nuclear magnetic resonance biomarker of systemic inflammation. Clin Chem.

[9] Akinkuolie AO, Buring JE, Ridker PM, Mora S (2014). A novel protein glycan biomarker and future cardiovascular disease events. JAHA.

[10] Akinkuolie AO, Glynn RJ, Ridker PM, Mora S (2014). Protein glycan side-chains, rosuvastatin therapy, and incident vascular events: an anlysis from the JUPITER trial. Circ.

[11] Muhlestein JB, Mays H, Winegar D, Rollo J, Connelly M, Otvos J, Anderson J (2014). GlycA and GlycB, novel NMR biomarkers of inflammation, strongly predict future cardiovascular events, but not the presence of coronary artery disease (CAD), among patients undergoing, coronary angiography: the Intermountain Heart Collaborative Study. JACC.

[12] McGarrah R, Craig D, Haynes C, Dowdy ZE, Shah S, Kraus W (2015). GlycA, a novel biomarker of systemic inflammation, improves cardiovascular risk prediction in a high-risk coronary catheterization cohort. JACC.

[13] Gruppen EG, Riphagen IJ, Connelly MA, Otvos JD, Bakker SJ, Dullaart RP (2015). GlycA, a pro-inflammatory glycoprotein biomarker, and incident cardiovascular disease: relationship with C-reactive protein and renal function. PLoS One.

[14] Akinkuolie AO, Pradhan AD, Buring JE, Ridker PM, Mora S (2015). Novel protein glycan side-chain biomarker and risk of incident type 2 diabetes mellitus. ATVB.

[15] Connelly MA, Gruppen EG, Wolak-Dinsmore J, Matyus SP, Riphagen IJ, Shalaurova I (2015). GlycA, a marker of acute phase glycoproteins, and the risk of incident type 2 diabetes mellitus: PREVEND study. Clin Chim Acta.

[16] Ormseth MJ, Chung CP, Oeser AM, Connelly MA, Sokka T, Raggi P (2015). Utility of a novel inflammatory marker, GlycA, for assessment of rheumatoid arthritis disease activity and coronary atherosclerosis. Arthritis Res & Ther.

[17] Amon R, Reuven EM, Leviatan Ben-Arye S, Padler-Karavani V (2014). Glycans in immune recognition and response. Carbohydr Res.

[18] Nagae M, Yamaguchi Y (2012). Function and 3D structure of the N-glycans on glycoproteins. Int J Mol Sci.

[19] Ogata S, Shimizu C, Franco A, Touma R, Kanegaye JT, Choudhury BP (2013). Treatment response in kawasaki disease is associated with sialylation levels of endogenous but not therapeutic intravenous immunoglobulin G. PLoS One.

[20] Lin J, Jain S, Sun X, Liu V, Sato YZ, Jimenez-Fernandez S (2014). Lipoprotein particle concentrations in children and adults following Kawasaki disease. J Pediatr.

[21] Chung CP, Oeser A, Raggi P, Sokka T, Pincus T, Solus JF (2010). Lipoprotein subclasses determined by nuclear magnetic resonance spectroscopy and coronary atherosclerosis in patients with rheumatoid arthritis. J Rheumatol.

[22] McInnes IB, Thompson L, Giles JT, Bathon JM, Salmon JE, Beaulieu AD (2013). Effect of interleukin-6 receptor blockade on surrogates of vascular risk in rheumatoid arthritis: MEASURE, a randomised, placebo-controlled study. Ann Rheum Dis.

[23] Jeyarajah EJ, Cromwell WC, Otvos JD (2006). Lipoprotein particle analysis by nuclear magnetic resonance spectroscopy. Clinics Lab Med.

[24] Chung CP, Ormseth MJ, Oeser A, Solus JF, Connelly MA, Otvos JD, Stein CM. GlycA, a novel NMR marker of inflammation, is elevated in systemic lupus erythematosus. Lupus. 2016;25:296–300. 10.1177/0961203315617842 PMC4767584 26637290

[25] G HB, Rao VS, Kakkar VV. Friend Turns Foe: Transformation of anti-inflammatory HDL to proinflammatory HDL during acute-phase response. Cholesterol. 2011:274629. doi:10.1155/2011/274629 10.1155/2011/274629 PMC3065911 21490770

[26] Jahangiri A (2010). High-density lipoprotein and the acute phase response. Curr Opin Endocrinol Diabetes Obes.

[27] Lahdesmaki K, Plihtari R, Soininen P, Hurt-Camejo E, Ala-Korpela M, Oorni K (2009). Phospholipase A(2)-modified LDL particles retain the generated hydrolytic products and are more atherogenic at acidic pH. Atherosclerosis.

[28] de la Llera Moya M, McGillicuddy FC, Hinkle CC, Byrne M, Joshi MR, Nguyen V (2012). Inflammation modulates human HDL composition and function in vivo. Atherosclerosis.

[29] Zannis VI, Fotakis P, Koukos G, Kardassis D, Ehnholm C, Jauhiainen M (2015). HDL biogenesis, remodeling, and catabolism. Handb Exp Pharmacol.

[30] Gabay C, Kushner I (1999). Acute-phase proteins and other systemic responses to inflammation. NEJM.

[31] Fraser CG (2004). Test result variation and the quality of evidence-based clinical guidelines. Clin Chim Acta.

[32] Westgard JO, Darcy T (2004). The truth about quality: medical usefulness and analytical reliability of laboratory tests. Clin Chim Acta.

