# Supplementary figures and images for: Differences in GlycA and lipoprotein particle parameters may help distinguish acute kawasaki disease from other febrile illnesses in children
Source: BMC Pediatr. 2016 Sep 5;16(1):151. doi: 10.1186/s12887-016-0688-5 (PMC5011873; doi:10.1186/s12887-016-0688-5)

## Slide 1
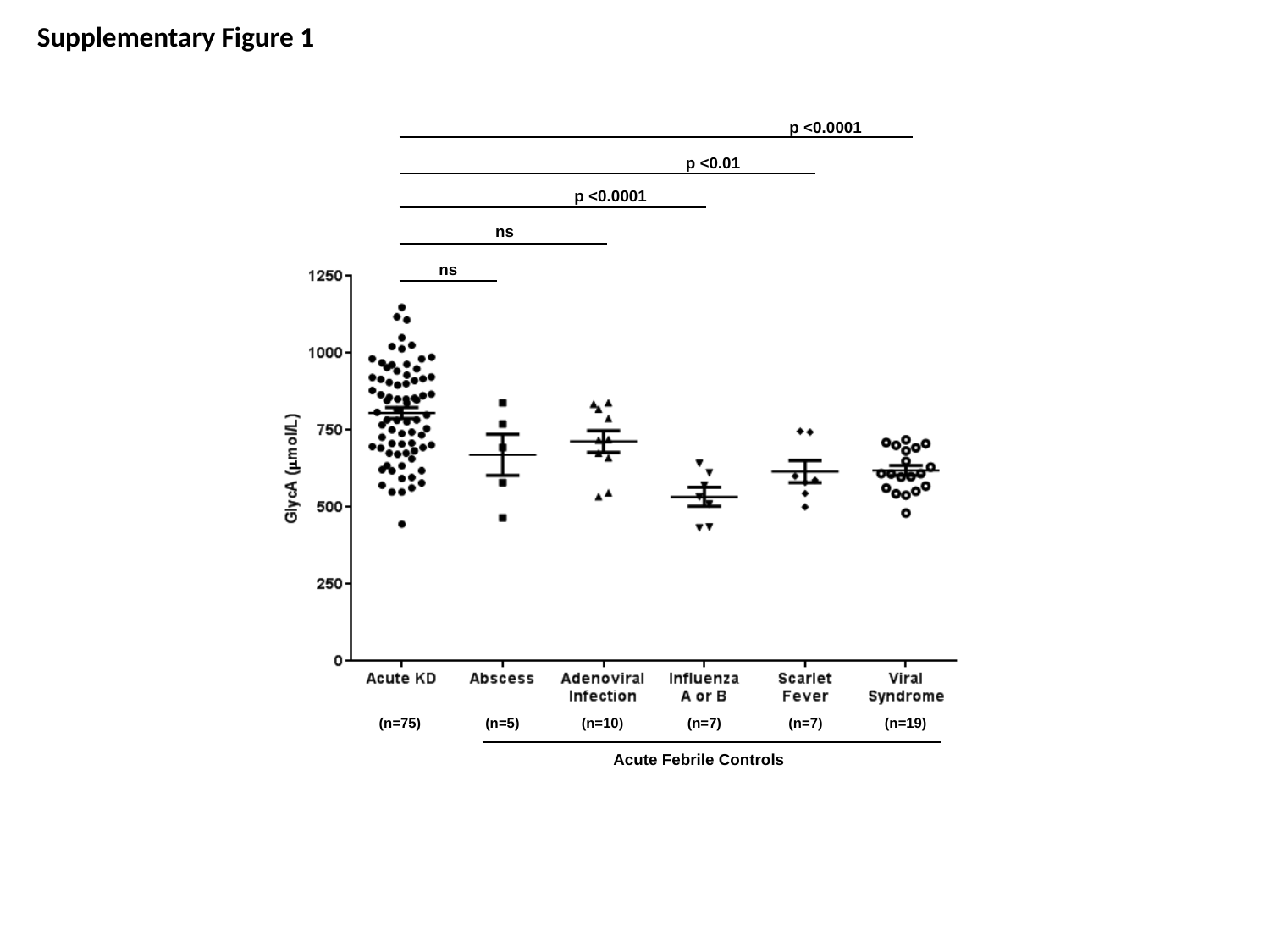

Supplementary Figure 1
p <0.0001
p <0.01
p <0.0001
ns
ns
(n=75)
(n=5)
(n=10)
(n=7)
(n=7)
(n=19)
Acute Febrile Controls

Supplement: Additional file 1: Figure S1. — Plasma concentrations of GlycA (μmol/L) in subjects with: acute KD, abscess, adenoviral infection, influenza A or B, scarlet fever or viral syndrome. ns = not statistically significant. Horizontal bars represent median and interquartile range. (PPTX 70 kb) [file 12887_2016_688_MOESM1_ESM.pptx]

## Slide 1
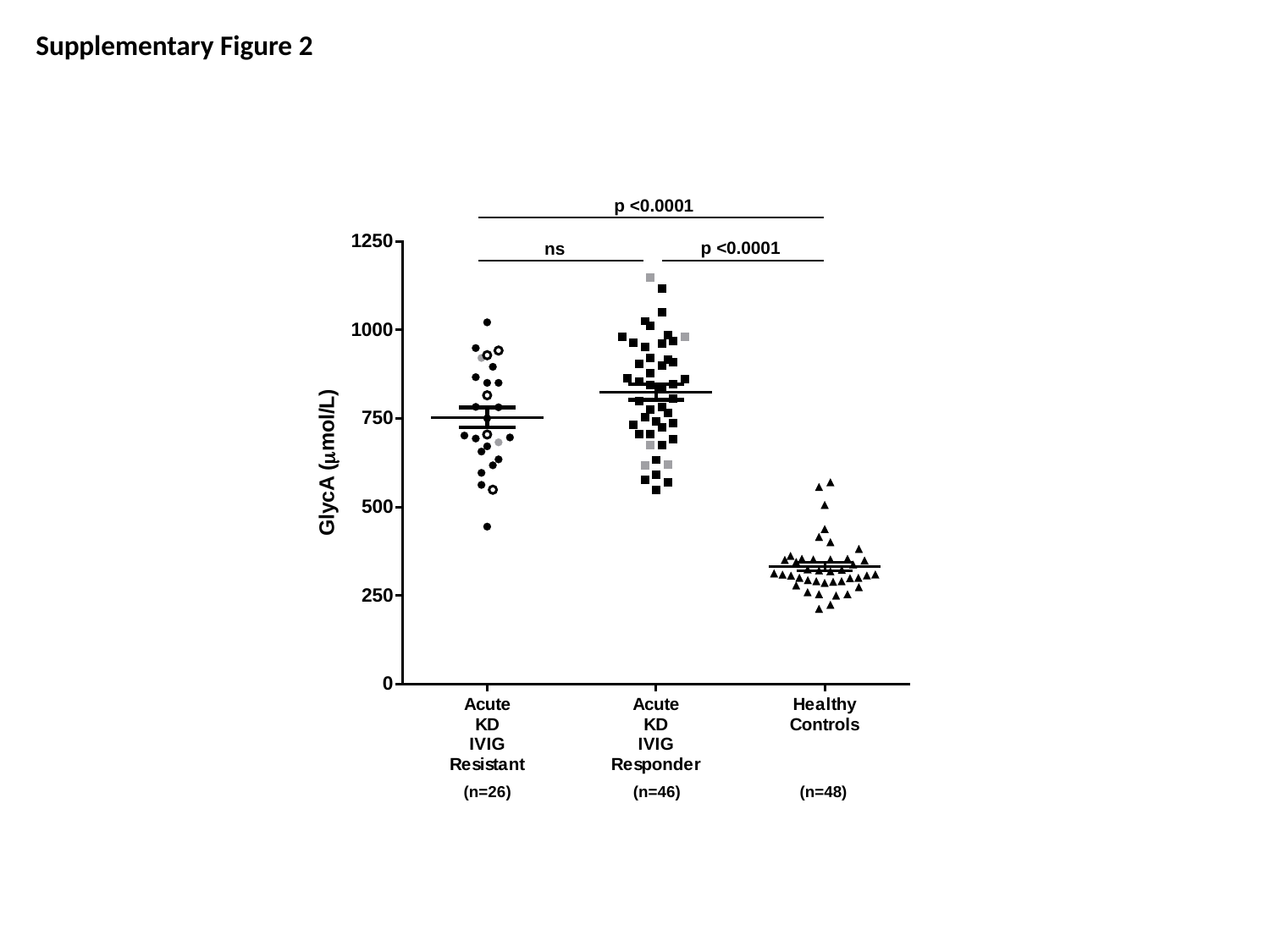

Supplementary Figure 2
p <0.0001
p <0.0001
ns
(n=26)
(n=46)
(n=48)

Supplement: Additional file 2: Figure S2. — Plasma concentrations of GlycA (μmol/L) in acute KD patients who responded to intravenous immunoglobulin (IVIG) treatment, those who were IVIG-resistant, and healthy controls. Noted are acute KD subjects with a normal echocardiogram (solid black symbol) as well as those who developed a CAA (open symbol) or a dilated coronary artery (solid gray symbol). ns = not statistically significant. Horizontal bars represent median and interquartile range. (PPTX 58 kb) [file 12887_2016_688_MOESM2_ESM.pptx]
