# Supplementary material for: Differences in GlycA and lipoprotein particle parameters may help distinguish acute kawasaki disease from other febrile illnesses in children
Source: BMC Pediatr. 2016 Sep 5;16(1):151. doi: 10.1186/s12887-016-0688-5 (PMC5011873; doi:10.1186/s12887-016-0688-5)
Supplement: Additional file 3: Table S1. — Correlations among inflammatory markers in acute KD subjects. (DOCX 15 kb) [file 12887_2016_688_MOESM3_ESM.docx]

**Supplementary Table 1. Correlations among inflammatory markers in acute KD subjects.**

|  | **GlycA** | **CRP** | **ESR** | **ANC** | **PMN** | **WBC** |
| --- | --- | --- | --- | --- | --- | --- |
| **WBC** | 0.43**^***^** | 0.16 | 0.32^**^ | 0.82**^****^** | 0.28^*^ | 1 |
| **PMN** | 0.18 | 0.11 | 0.17 | 0.57**^****^** | 1 | 0.28^*^ |
| **ANC** | 0.42**^***^** | 0.41**^***^** | 0.35^**^ | 1 | 0.57^****^ | 0.82**^****^** |
| **ESR** | 0.42**^***^** | 0.30^*^ | 1 | 0.35^**^ | 0.17 | 0.32^**^ |
| **CRP** | 0.26^*^ | 1 | 0.30^*^ | 0.41**^***^** | 0.11 | 0.16 |
| **GlycA** | 1 | 0.26^*^ | 0.42**^***^** | 0.42**^***^** | 0.18 | 0.43**^***^** |
| **Illness day** | 0.48**^****^** | -0.35^**^ | -0.03 | 0.005 | -0.02 | 0.17 |

^*^p <0.05, ^**^p <0.01; **^***^**p <0.001; **^****^**p <0.0001. WBC, white blood cell count; PMN, polymorphonuclear cells; ANC, absolute neutrophil count; ESR, erythrocyte sedimentation rate; CRP, C-reactive protein; GlycA, NMR-measured marker of systemic inflammation.
