# Supplementary material for: Differences in GlycA and lipoprotein particle parameters may help distinguish acute kawasaki disease from other febrile illnesses in children
Source: BMC Pediatr. 2016 Sep 5;16(1):151. doi: 10.1186/s12887-016-0688-5 (PMC5011873; doi:10.1186/s12887-016-0688-5)
Supplement: Additional file 4: Table S2. — SPerformance characteristics for GlycA, lipoprotein parameters, CRP and ESR restricted to illness days 6-10. (DOCX 23 kb) [file 12887_2016_688_MOESM4_ESM.docx]

**Supplementary Table 2. Performance characteristics for GlycA, lipoprotein parameters, CRP and ESR restricted to illness days 6-10.**

| **Test** | **AUC** | **95% CI** | **Sensitivity** | **Specificity** | **Positive LR** | **Negative LR** | **Wald Test**  **z-statistic** | **PPV** | **NPV** |
| --- | --- | --- | --- | --- | --- | --- | --- | --- | --- |
| CRP | 0.730 | 0.587-0.873 | 0.556 | 0.938 | 8.89 | 0.47 | 3.15 | 0.94 | 0.55 |
| ESR | 0.879 | 0.778-0.979 | 0.857 | 0.864 | 6.29 | 0.17 | 7.37 | 0.91 | 0.78 |
| LDL-P/HDL-P | 0.842 | 0.732-0.952 | 0.846 | 0.870 | 6.49 | 0.18 | 6.09 | 0.92 | 0.77 |
| GlycA | 0.891 | 0.814-0.967 | 0.667 | 0.957 | 15.33 | 0.35 | 9.99 | 0.96 | 0.63 |
| GlycA + LDL-P | 0.909 | 0.823-0.994 | 0.718 | 0.957 | 16.51 | 0.29 | 9.40 | 0.97 | 0.67 |
| GlycA + LDL-P/HDL-P | 0.910 | 0.841-0.979 | 0.692 | 0.957 | 15.92 | 0.32 | 11.65 | 0.96 | 0.65 |
| Total LDL-P | 0.864 | 0.765-0.963 | 0.615 | 0.957 | 14.15 | 0.14 | 7.22 | 0.96 | 0.59 |
| IDL-P | 0.580 | 0.435-0.726 | - | - | - | - | - | - | - |
| Large LDL-P | 0.675 | 0.537-0.813 | - | - | - | - | - | - | - |
| Small LDL-P | 0.784 | 0.665-0.902 | - | - | - | - | - | - | - |
| Total HDL-P | 0.691 | 0.556-0.826 | - | - | - | - | - | - | - |
| Large HDL-P | 0.514 | 0.353-0.674 | - | - | - | - | - | - | - |
| Medium HDL-P | 0.558 | 0.411-0.705 | - | - | - | - | - | - | - |
| Small HDL-P | 0.733 | 0.605-0.861 | - | - | - | - | - | - | - |
| Total VLDL-P | 0.583 | 0.434-0.732 | - | - | - | - | - | - | - |
| Large VLDL-P | 0.638 | 0.496-0.779 | - | - | - | - | - | - | - |
| Medium VLDL-P | 0.563 | 0.414-0.712 | - | - | - | - | - | - | - |
| Small VLDL-P | 0.614 | 0.471-0.756 | - | - | - | - | - | - | - |

Diagnostic indices calculated at the following thresholds corresponding to the highest Positive likelihood ratio and Odds ratio: CRP 5.4 mg/dL; ESR 44 mm/h; LDL-P/HDL-P ratio 65, GlycA 834 µmol/L, GlycA + LDL-P 2204; GlycA + LDL-P/HDL-P ratio 910, Total LDL-P 1398 nmol/L. AUC, area under the curve; CI, confidence intervals; CRP, C-reactive protein; ESR, erythrocyte sedimentation rate; HDL-P, high density lipoprotein particle number; IDL-P, intermediated density lipoprotein particle number; LDL-P, low density lipoprotein particle number; LR, likelihood ratio; NPV, negative predictive value; PPV, positive predictive value; VLDL-P, very low density particle number.
